# Diversity of Insect Sesquiterpenoid Regulation

**DOI:** 10.3389/fgene.2020.01027

**Published:** 2020-09-10

**Authors:** Stacey S. K. Tsang, Sean T. S. Law, Chade Li, Zhe Qu, William G. Bendena, Stephen S. Tobe, Jerome H. L. Hui

**Affiliations:** ^1^Simon F.S. Li Marine Science Laboratory, State Key Laboratory of Agrobiotechnology, School of Life Sciences, The Chinese University of Hong Kong, Hong Kong, China; ^2^Department of Biology, Queen’s University, Kingston, ON, Canada; ^3^Department of Cell and Systems Biology, University of Toronto, Toronto, ON, Canada

**Keywords:** insect, sesquiterpenoid, juvenile hormone, microRNA, evolution

## Abstract

Insects are arguably the most successful group of animals in the world in terms of both species numbers and diverse habitats. The sesquiterpenoids juvenile hormone, methyl farnesoate, and farnesoic acid are well known to regulate metamorphosis, reproduction, sexual dimorphism, eusociality, and defense in insects. Nevertheless, different insects have evolved with different sesquiterpenoid biosynthetic pathway as well as products. On the other hand, non-coding RNAs such as microRNAs have been implicated in regulation of many important biological processes, and have recently been explored in the regulation of sesquiterpenoid production. In this review, we summarize the latest findings on the diversity of sesquiterpenoids reported in different groups of insects, as well as the recent advancements in the understanding of regulation of sesquiterpenoid production by microRNAs.

## Diverse Biosynthetic Pathways and Types of Insect Sesquiterpenoids

**FIGURE 1:**
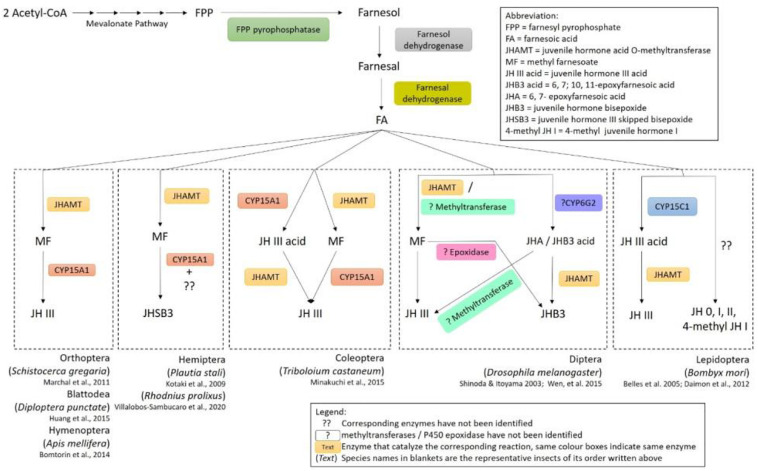
Diverse biosynthetic pathways of juvenile hormones in insects.

In insects and crustaceans, sesquiterpenoid hormones including farnesoic acid (FA), methyl farnesoate (MF) and juvenile hormone (JH) regulate the development, metamorphosis and reproduction (Cheong et al., 2015). The beginning step in the biosynthesis of the sesquiterpenoids starts from acetyl-CoA which goes through the universal eukaryotic mevalonate (MVA) pathway to synthesize farnesyl pyrophosphate (FPP) (Tobe and Bendena, 1999; Belles et al., 2005; Hui et al., 2010, 2013). In the presence of FPP pyrophosphatase, FPP is then converted to farnesol and can further generate farnesal with the catalyzation by farnesol dehydrogenase. Farnesoic acid (FA) will then be generated via further dehydrogenation with farnesal dehydrogenase in different insects. A summary of the sesquiterpenoid biosynthetic pathway is shown in Figure 1.

Despite all insects utilizing a common biosynthetic pathway in the production of FA, diverse pathways have evolved in the downstream process of sesquiterpenoids production. For insects in the order blattodea, coleoptera, diptera, and orthoptera, esterification of FA occurs in the corpora allata (CA), which will form MF catalyzed by a *SAM*-dependent juvenile hormone acid *O-*methyltransferase (JHAMT) (Shinoda and Itoyama, 2003). In insects such as cockroaches (Huang et al., 2015), honeybees (Bomtorin et al., 2014), locusts (Marchal et al., 2011), and pea aphids (Daimon and Shinoda, 2013), MF is oxidized by epoxidase CYP15A1 in formation of JH-III (Figure 1). Direct applications of FA on fruit flies increased the biosynthesis of MF and JH-III in both larval and adult stages, while JHB3 biosynthesis is inhibited in larvae (Bendena et al., 2011). Moreover, diverse biosynthetic pathways for production of JH-III have also been identified in other insects (Figure 1). For instance, in the coleopterans such as beetles, CYP15A1 can first oxidize FA to form JH-III acid, followed by methylation with JHAMT resulting in the formation of JH-III (Minakuchi et al., 2015; Jiang et al., 2017); while in lepidopterans, the conversion of FA to JH-III acid is performed with another epoxidase CYP15C1 followed by subsequent methylation by JHAMT (Daimon et al., 2012; Figure 1). Furthermore, different sesquiterpenoid products have also been identified in various types of insects (Figure 1 and Table 1). In the dipterans including flies, JH-III bisepoxide (JHB3) has been identified (Richard et al., 1989). In the hemipterans like the stinkbugs, JH-III skipped bisepoxide (JHSB3) is formed (Kotaki et al., 2009); and in the lepidopterans such as moths, specific JH homologs including JH-I, JH-II, JH-0, and 4-methyl JH-I are produced (Belles et al., 2005; Figure 1 and Table 1). It is worth mentioning that JH-I is found in the male accessory glands of the cecropia moth, and whether it performs the suspected hormonal function remains unknown (Paroulek and Sláma, 2014; De Loof and Schoofs, 2019).

**TABLE 1:** Different types of juvenile hormones isolated from hexapods.

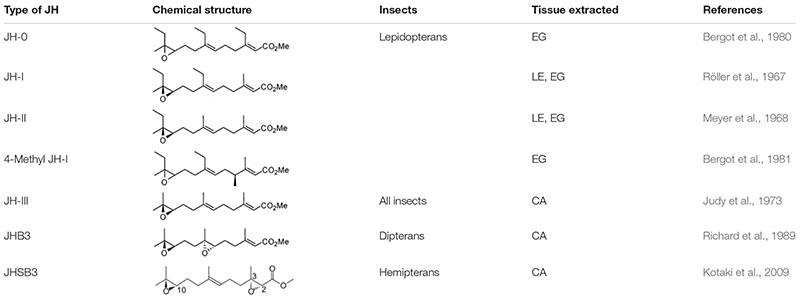

*CA, corpora allata; EG, egg; LE, lipid extract.*

## Diverse Roles of Sesquiterpenoids in Insects

### Regulation of Metamorphosis

**FIGURE 2:**
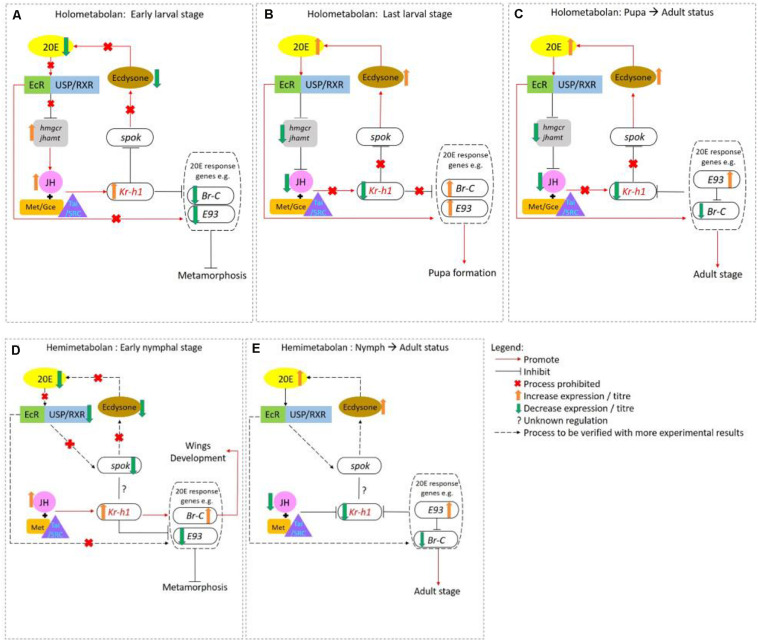
Interaction of sesquiterpenoid juvenile hormone (JH) and ecdysteroid during metamorphosis in holometabolans **(A–C)** and hemimetabolans **(D,E)**. **(A)** In early larval stages of holometabolous insects, JH-Met/Tai receptor complex activates the transcription of primary JH-early responsive gene *Kr-h1* which prevents immature larvae from precocious larval development by inhibiting *Br-C*, *E93*, and *Spok* expression. **(B)** When JH levels drastically drop in the last larval stage, 20E acts through EcR/USP to activate the transcription of 20E-early responsive genes such as *Br-C*, *E93, E74*, *E75*, *Ftz*-*f1* and initiate larval-pupal transition. **(C)** At the end of the pupal stage, the *Br-C* levels decline again which upregulates the expression of *E93* that drives the pupa-adult transition. In hemimetabolous insect, JH titer remains high from hatching until the last nymphal stage. **(D)** During the early nymphal, high *Kr-h1* expression level is maintained by JH which inhibits metamorphosis by repressing *E93* expression. **(E)** In the last nymphal stage, the JH titers fall followed by the *Kr-h1* expression level. For details, please refer to main text and Truman, 2019.

A special feature of insects is that they have evolved with distinct modes of metamorphosis, including hemimetaboly (incomplete) and holometaboly (complete) (Sehnal et al., 1996). These biological events are collectively controlled by sesquiterpenoids that inhibit metamorphosis, and ecdysteroids such as 20-hydroxyecysone (20E) that trigger metamorphosis (Konopova et al., 2011; Liu et al., 2018; Niwa and Niwa, 2014a,b). In general, sesquiterpenoid inhibits ecdysteroids action, and when their biosynthesis in the CA is suppressed via the inhibition of JHAMT and 3-hydroxy-3-methylglutaryl Coenzyme-A reductase (HMGR), metamorphosis can then occur (Cheong et al., 2015; Liu et al., 2018; Qu et al., 2018). An overview is shown in Figure 2.

**FIGURE 3:**
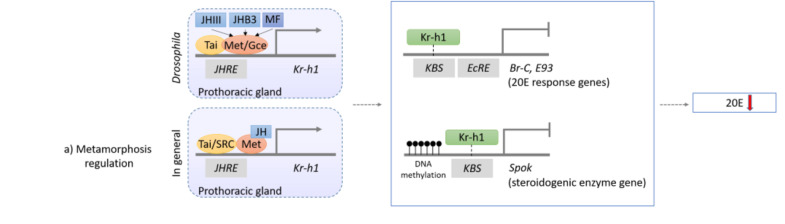
Schematic diagram showing the mechanism of sesquiterpenoids in metamorphosis regulation in *Drosophila* and other insects. In fly *Drosophila*, JH-III, JHB3, and MF will bind to the JH receptor Met or Gce, while in other insects, JH-III will bind to Met in other insects (for details, please refer to text). The complex will then further dimerize with Tai and bind to specific JHRE to initiate the expression of *Kr-h1*. Kr-h1 protein will then bind to the KBS to inhibit expressions of 20E response genes (*Br-C* and *E93*), and will also bind to KBS and initiates DNA methylation of a steroidogenic enzyme gene *Spok*, which will all result in the lower titer of 20E and inhibition of metamorphosis.

In the best studied holometabolous insect, the fly *Drosophila melanogaster*, sesquiterpenoids exert *status quo* function to prevent metamorphosis in the early larval stage (Cheong et al., 2015; Qu et al., 2018). Sesquiterpenoids JH-III, JHB3, and their immediate precursor MF can all bind to the *C*-terminal of the intracellular receptor Methoprene-tolerant (Met) or its paralog named Germ-cell expressed (Gce) in *Drosophila*, which encodes a transcription factor of the bHLH-PAS family (Ashok et al., 1988; Jindra et al., 2015; Wen et al., 2015). The binding affinities of sesquiterpenoids to Gce are differ with a rank order of JH-III > JHB3 > MF which is in line with their developmental potency (Bittova et al., 2019). After the binding of JH with Met or Gce in formation of a functional complex, another bHLH-PAS protein that acts as the steroid receptor co-activator [Taiman (Tai)] in *D. melanogaster* or SRC in other insect species is recruited, which together binds to the specific JH response element (JHRE) on the promoter region of *Krüppel homolog 1* (*Kr-h1*) to activate transcription (Kayukawa et al., 2012; Qu et al., 2018). Previous studies have demonstrated that *Kr-h1* can transduce the JH signal to repress 20E primary responsive genes, including *ecdysone receptor* (*EcR*), *Broad-complex* (*Br-C*), ecdysone-inducible proteins *E75* and *E93*, which subsequently inhibit 20E biosynthesis in the prothoracic gland (Kayukawa et al., 2016; Liu et al., 2018); and can also inhibit the expression of steroidogenic enzyme gene *Spok* by binding to the Kr-h1 binding site (KBS) and turn on the methylation which in turns also leads to the suppression of ecdysone biosynthesis (Song and Zhou, 2019; Zhang T. et al., 2018; Figures 2, 3).

In other holometabolous insects including beetle *Tribolium castaneum*, moths *Bombyx mori* and *Helicoverpa armigera*, as well as hemimetabolous insects including cockroach *Blattella germanica*, planthopper *Nilaparvata lugens*, and stinkbug *Pyrrhocoris apterus* and *Rhodnius prolixus*, *Kr-h1* has also exhibited anti-metamorphic effects (Minakuchi et al., 2009; Konopova et al., 2011; Lozano and Belles, 2011; Kayukawa et al., 2017; Li et al., 2018; Zhang W. N. et al., 2018).

During the larval-pupal transition in *Drosophila*, 20E binds to EcR proteins and Ultraspiracle (Usp) to form a heterodimer (Riddiford et al., 2000), and this complex will further trigger the transcription of 20E primary-response genes including *Br-C*, *E74*, *E75*, and *E93*. These downstream genes have been identified with essential functions in molting. For instances, E93 enables the larval tissues to execute apoptosis and promotes the formation of adult tissues (Ureña et al., 2016); and the Gce/Tai (but not Met/Tai) complex activates E75A functions in preimaginal molts (Dubrovsky et al., 2011). In beetle *T. castaneum*, Met has also proven to bind JH with high affinity via the highly conserved hydrophobic pocket within its PAS-B domain (Charles et al., 2011). In lepidopteran, USP can also bind JH (Dubrovsky, 2005). In moth *Manduca*, JP29 isolated from epidermis has also been suggested as another potential JH receptor, which has found to be highly specific to JH binding but with low affinity (Truman and Riddiford, 2002).

### Regulation of Reproduction

Apart from repressing metamorphosis in insects, sesquiterpenoids also play an important role in stimulating reproduction in adult insects, including processes such as vitellogenesis, oogenesis and polyploidization (Wyatt and Davey, 1996). In female *Drosophila*, sesquiterpenoids have long been known to regulate the oogenesis and vitellogenesis (Postlethwait and Weiser, 1973; Swevers et al., 2005; Riddiford, 2012). The titer of JH is promoted with expression of ecdysis triggering hormone (ETH) binding to its receptor (ETHR) whose synthesis is governed by 20E (Meiselman et al., 2017; Roy et al., 2018).

**FIGURE 4:**
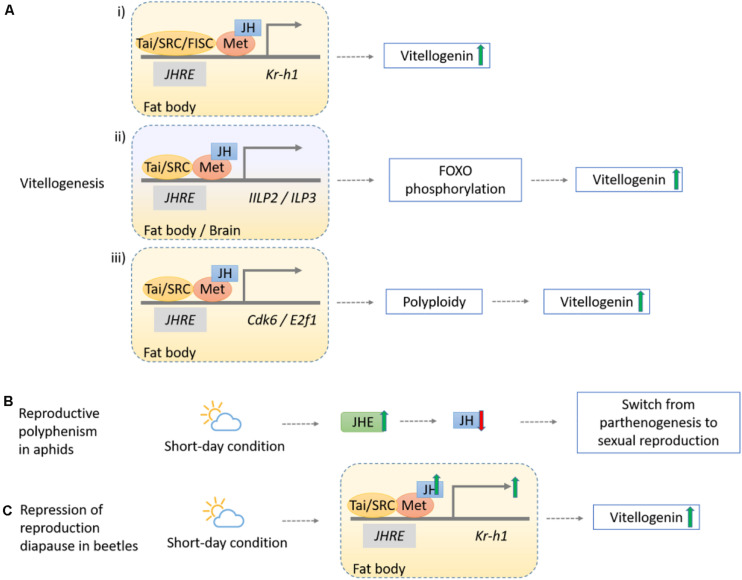
Schematic diagram showing the mechanisms of sesquiterpenoids in regulation of insect reproduction**. (Ai)** The JH-Met-Tai/SRC complex upregulates Kr-h1 to increase Vg synthesis level, as observed in *T. castaneum*, *A. aegypti* (with an additional complex FISC), *L. migratoria* but not in *P. apterus*. **(Aii)** The JH-Met-Tai complex initiates transcriptions of *ILP2* and *ILP3*, which phosphorylates the fork head transcription factor (FOXO) through ILP signaling pathway and induces *Vg* expression in *T. castaneum*. **(Aiii)** The JH-Met-Tai/SRC complex promotes expression of core mediators in cell cycle progression, *Cdk6* and *E2f1*, to facilitate vitellogenesis in *L. migratoria*. **(B)** Reproductive polyphenism in aphid *A. pisum* occurs during the short-day condition given the increased JHE activity, and the lowering of JH result in the switch from parthenogenesis to sexual reproduction. **(C)** Repression of reproduction diapause in beetles *C. bowringi* initiates in short-day condition where the upregulation of the JH-Met-Kr-h1 pathway genes expression increases Vg synthesis.

Similar but diverse mechanisms have also been discovered in other insects. In the beetle *T. castaneum*, JH-mediated *Met* and *Kr-h1* promote vitellogenin (Vg) synthesis in the fat body (Parthasarathy et al., 2010; Figure 4Ai), and *Met* can also trigger insulin-like peptides (ILPs) *ILP2* and *ILP3* by AKT pathway to phosphorylate the fork head transcription factor (FOXO) and induce *Vg* expression (Sheng et al., 2011; Figure 4Aii). In mosquito *Aedes aegypti*, expression of *Kr-h1* triggered by Met together with Cycle and steroid receptor coactivator SRC/FISC after adult emergence supported that sesquiterpenoid is essential for previtellogenic development (Zhu et al., 2010; Shin et al., 2012). In migratory locust *Locusta migratoria*, JH together with Met/SRC complex are found to be pivotal in maintaining *Vg* expression and oocyte development (Song et al., 2014), and can promote cell polyploidization by regulating the expression of *cyclin-dependent kinase 6* (*Cdk6*) and *adenovirus E2 factor-1* (*E2f1*) (Wu et al., 2016; Wu Z. et al., 2018; Figure 4Aiii). JH activates Na^+^/K^+^-ATPase for the induction of patency in vitellogenic follicular epithelium, where Vg can then reach the surface of maturing oocyte (Jing et al., 2018). In the stinkbug *P. apterus*, nevertheless, Vg synthesis is mainly regulated by JH signaling genes *Met* and *Tai* independent of Kr-h1 (Smykal et al., 2014).

In addition, sesquiterpenoids can mediate insect reproduction under different light conditions. In aphids, reproductive polyphenism alternates their reproductive modes from parthenogenesis to sexual reproduction given different photoperiodic duration. In *Acyrthosiphon pisum*, enhanced sesquiterpenoid degradation by juvenile hormone esterase (JHE) accounts for the lower JH titer during short-day conditions that produces sexual morphs, in contrast to the higher JH titer in parthenogenetic morphs during long-day conditions (Ishikawa et al., 2012; Figure 4B). In beetle *Colaphellus bowringi*, high sesquiterpenoid titer upregulates expression of vitellogenin receptor (VgR) via JH-Met-Kr-h1 signaling and promotes Vg synthesis and ovary development during short-day period, while low JH titer initiates reproductive diapause and promotes lipid storage in the fat body instead of Vg synthesis during the long-day period (Liu et al., 2016, 2019; Figure 4C).

### Sexual Dimorphism and Dimorphic Behavior

**FIGURE 5:**
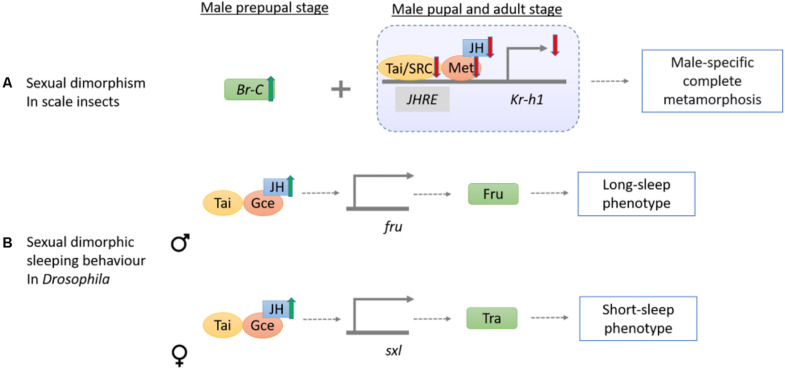
Schematic diagram showing the mechanisms of sesquiterpenoids in regulation of insect sexual dimorphism and dimorphic behavior. **(A)** In scale insects, sexual dimorphism of winged male adults is linked to the signaling of JH, Met, Tai/SRC, and *Kr-h1*, whereas they are increased in early developmental stages but decreased during the pupal and adult stages. **(B)** The sexual dimorphic sleeping behavior in *Drosophila* is maintained by the JH-Gce signal on the *fru* and *sxl* genes in male and female, respectively. *fru* then encodes Fru for inducing long-sleep pattern in male while *sxl* induces Tra for short-sleep phenotype in female.

Sexual dimorphism is commonly observed in insects. Nevertheless, the extreme sexually dimorphic traits of juvenile-like females without pupation and ephemeral winged males after a pupal stage in scale insects have raised questions as to how these features could arise. By transcriptomic and qRT-PCR analyses of post-embryonic stages of *Ericerus pela*, lower *Met*, *Tai*, and *Kr-h1* expression levels are found in pupal and adult males as compared to females. Together with a surge in *Br-C* expression in male prepupal stage, the sex-specific regulation lead to the complete metamorphosis in males but not in females (Yang et al., 2015; Figure 5A). In another scale insect *Planococcus kraunhiae*, qRT-PCR analysis on a daily sampling of different development stages reveal that expression levels of *Kr-h1* are higher in male-biased embryos and early nymphs, and lower during prepupal and after pupal stages (Vea et al., 2016). However, elevation of JH or *Met*, *Tai*, and *Kr-h1* gene expressions as observed in *E. pela* is not found in the adult *P. kraunhiae* females.

In *Drosophila*, JH can also control sexual dimorphic behaviors including locomotory and sleeping activities (Belgacem and Martin, 2007; Wu B. et al., 2018; Figure 5B). In the presence of JH by overexpression of *JHAMT*, longer sleep in males and shorter sleep in females are observed (Wu B. et al., 2018). Interestingly, *gce* mutant male flies sleep less while female sleep more but mutation in the *Met* dose not exhibit a similar result (Wu B. et al., 2018). The binary switch gene *sex-lethal* (Sxl) can impose female development via promoting expression of *fruitless* (*fru*), *doublesex* (*dsx*), and *transformer* (*tra*). Male development occurs when sxl is turned off (Kappes et al., 2011). In the *jhamt* and *gce* mutant, Fru, sxl, and tra transcript level were almost halved. Decreasing sleep time occurred when *fru* in male flies and when female *tra* was expressed in Fru neurons of males, suggesting JH-Gce signaling can potentially act as a regulatory pathway in sexually dimorphic sleep pattern (Wu B. et al., 2018).

### Eusociality

Some insects such as ants, bees, termites and wasps are well known for their eusociality in which they live cooperatively in a colony and only some individuals are reproductive. Such processes have also been linked to JH.

**FIGURE 6:**
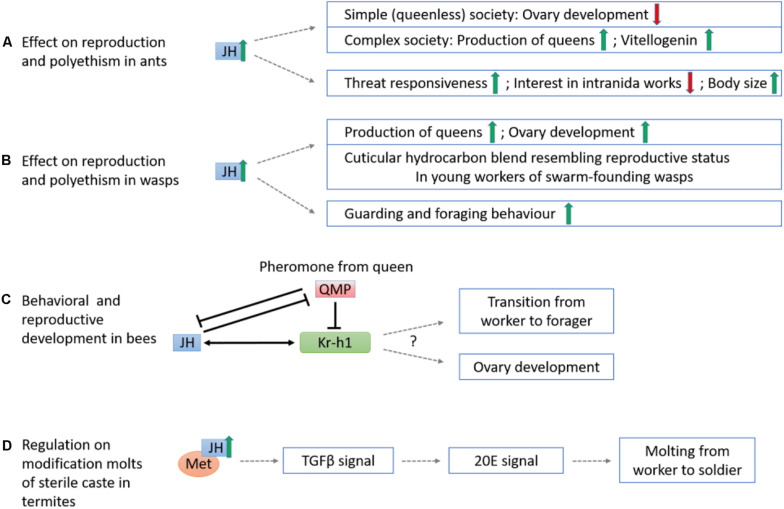
Schematic diagram showing the mechanisms of sesquiterpenoids in regulation of insect eusociality. **(A)** The effect of JH on reproduction and polyethism in ants. The elevation of JH represses ovary development in simple, queenless societies but promotes queen production and vitellogenin expression in complex societies. JH also induces threat responsiveness and reduces interest in intraida works in ants, and may also increase their body size. **(B)** The effect of JH on reproduction and polyethism in wasps. JH generally stimulates the production of queens and ovary development, and mediates cuticular hydrocarbon blend resembling reproductive status in young workers of swarm-founding wasps. Besides, JH triggers the guarding and foraging behavior. **(C)** The queen releases QMP which downregulates JH and *Kr-h1* and thus inhibits ovary development of workers and the transition from in-hive worker to forager in bees. **(D)** The increase of JH in JH-Met signaling pathway induces TGFβ and 20E signals that promote the modification molts from worker to soldier in the sterile caste of termite *Z*. *nevadensis*.

Across ant species, the effects of JH act with different eusocial complexity (Figure 6A). For ants with simple, queenless societies, e.g., *Streblognathus* and *Diacamma*, low JH titer is recorded in the gamergates with high individual ranks within the hierarchy, and elevated JH level result in a loss of the reproductive status of the alpha workers (Sommer et al., 1993; Cuvillier-Hot et al., 2004; Brent et al., 2006). For species that have secondarily revert to queenless, simple societies, e.g., *Dinoponera quadriceps*, JH application can increase the regressed ovaries in queenless ants (Norman et al., 2019). For ants with complex society such as *Pogonomyrmex rugosus*, JH analogs (methoprene) stimulate the production of queens and upregulate *Vg* gene expression. The effect of JH in ants is interpreted as mimicking the effect of hibernation (Libbrecht et al., 2013), where low temperature or the associated photoperiod changes up-regulate the insulin/insulin-like growth factor signaling pathway (IIS) genes in queens. No direct result has proven the relationship of IIS and JH in ants to date, and yet, the production of JH in the CA is affected by the release of neuropeptides regulated by IIS in *Drosophila* (Tu et al., 2005). JH may also directly or indirectly regulate of caste polyethism via changing the division of labor and maternal effects. Elevated JH titer can alter the behavior of workers of *Acromyrmex octospinosus* leaf-cutting ants by making them more active, threat responsive, and less interested in intranida works such as taking care of larva and fungal cultivation (Norman and Hughes, 2016). During the maternal stage of *Pogonomyrmex* harvest ants, additional JH also resulted in a 50% increase in worker body size and significantly reduced in total number of progeny reared (Cahan et al., 2011).

Similarly, JH also appears to have different effects on wasp species with various eusociality (Figure 6B). Previous studies indicated JH could modulate age polyethism and promote the production of foragers in highly eusocial species such as *Polybiine* wasps (O’Donnell and Jeanne, 1993; O’Donnell, 1998), and could mediate both age polyethism (Shorter and Tibbetts, 2009) and reproductive division of labor in primitively eusocial species such as *Polistes*. Application of JH analog methoprene promotes the onset of guarding behavior, the number of foraging females, and stimulates the production of queens (Barth et al., 1975; Röseler et al., 1980, 1984, 1985; Lozano et al., 2015; Giray et al., 2005). Nevertheless, in other primitive eusocial species such as *Ropalidia marginata* that has both post-imaginal regulation of reproductive division of labor and age polyethism, JH could only accelerate ovarian development but not age polyethism (Agrahari and Gadagkar, 2003). For caste-flexible swarm-founding wasp *Synoeca surinama*, JH functions as gonadotropin and directly modifies the cuticular hydrocarbon blend of young workers to resemble that of a reproductive one but does not necessarily link to dominance behavior (Kelstrup et al., 2014).

It is worth also noting that the response to JH could be different among members of the same colony. In *Polistes canadensis*, the effect of JH on ovaries are different between queens and workers as a potential trophic advantage of the queens over the workers (Giray et al., 2005), while in *Polistes dominulus* where queens nest cooperatively with other queens, JH has a stronger effect on the dominance, fertility, and aggressiveness of large queens (Tibbetts and Izzo, 2009; Tibbetts et al., 2011, 2018). In species *Polistes metricus* with non-cooperative nest-founding queen pattern, JH leads to an increase of fertility for all individuals, but among the cooperative workers, large workers increase their fertility in response to JH more while small workers do not (Tibbetts and Sheehan, 2012).

In honeybees *Apis mellifera*, repression of ovary development, of in-hive workers, were induced by the downregulation of *Kr-h1* expression controlled by the queen’s release of mandibular pheromone (QMP) (Grozinger and Robinson, 2007; Figure 6C). In methoprene (JH analog)-treated workers, *Kr-h1* expression is no longer repressed by QMP suggesting an antagonistic relationship between sesquiterpenoids and QMP. In addition, the transition of working to foraging behavior were also found to link to a higher JH titer and *Kr-h1* level (Grozinger and Robinson, 2007). On the other hand, in the bumblebee *Bombus terrestris*, similar to the honeybee mentioned above, QMP reduces *Kr-h1* level but the difference in *Kr-h1* expression between the working and foraging bees are not significant (Shpigler et al., 2010). However, among a group of queenless workers, the dominant individuals have a higher *Kr-h1* expression with active ovaries whereas subordinate individuals have a downregulated *Kr-h1* expression level with undeveloped ovaries (Shpigler et al., 2010). These studies highlighted the possible roles of sesquiterpenoids in the eusociality in bees.

In termites, eusociality is maintained through differentiation into reproductive caste and sterile soldier caste, in which a higher JH titer induces differentiation of workers via an intermediate presoldier stage to become sterile soldiers (Roisin, 1996). Transcriptomic and RNA interference (RNAi) analyses in three molting stages (worker, presoldier and soldier) of termite *Zootermopsis nevadensis* show that the JH-Met and transforming growth factor beta (TGFβ) pathways are involved in the ecdysteroid synthesis for molting in soldier formation (Masuoka et al., 2018; Figure 6D). However, suppression on *Kr-h1* via RNAi has no effect on JH analog induced molting, demonstrating that the molting effect mainly depends on JH-Met induced pathways (Masuoka et al., 2018). This in turn also suggested that JH may alternatively promotes molting instead of solely inhibiting metamorphosis.

### Defense

**FIGURE 7:**
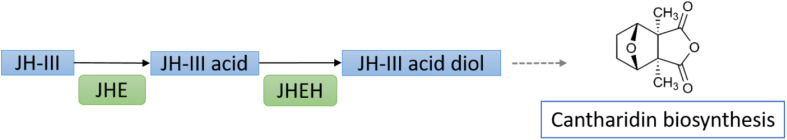
Schematic diagram showing the potential involvement of juvenile hormone in regulation of insect defensive toxin production. The metabolism of JH-III through its degradation pathway by JHE and JHEH is essential for the biosynthesis of the defensive toxin cantharidin in blister beetles.

Terpenes in plants have been the major focus on the understanding the plant defense against the insects, and the role of sesquiterpenoids in insect defense has also been documented in a much lesser extent when comparing to the aforementioned roles. In blister beetles, sesquiterpenoid cantharidin is produced and released as a defensive toxin during disturbance (Carrel et al., 1993). Transcriptomic analyses on *Mylabris cichorii* identified that the mevalonate pathway in synthesis of JH is correlated with the cantharidin biosynthesis (Huang et al., 2016). In another blister beetle *Epicauta chinensis*, RNAi knockdown of *CYP15A1* and JH epoxide hydrolase (JHEH) result in inhibition of cantharidin biosynthesis, suggesting degradation of JH-III is essential in producing potential precursors of cantharidin (Jiang et al., 2017; Figure 7).

## MicroRNA Regulations on Sesquiterpenoids

**FIGURE 8:**
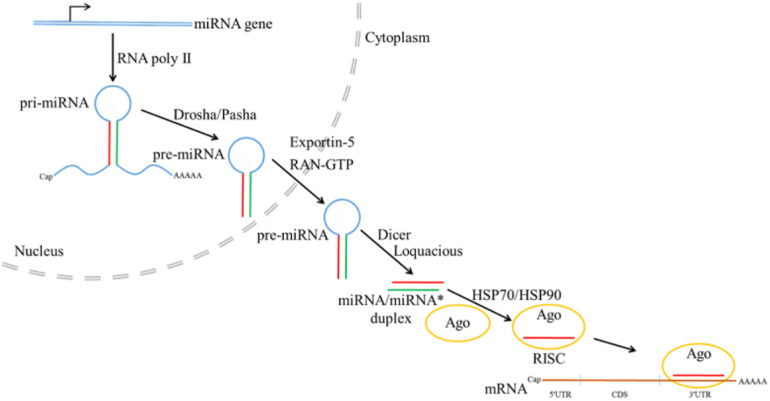
Canonical microRNA biogenesis pathway in *Drosophila* (the figure is summarized from Bartel, 2004; Denli et al., 2004; Kim et al., 2009; Iwasaki et al., 2010; Cao et al., 2017; Qu et al., 2018).

Non-coding RNAs such as microRNAs (miRNAs) have been implicated in regulation of many important biological processes (Lucas and Raikhel, 2013; Wang et al., 2014; Yang et al., 2014; Cao et al., 2017; Qu et al., 2018). In canonical miRNA biogenesis pathway in insects (Figure 8), primary miRNA transcript (pri-miRNA) is first transcribed from miRNA gene by RNA polymerase II, followed by processing by Drosha with the help of partner Pasha to generate the precursor miRNA (pre-miRNA) (Denli et al., 2004; Kim et al., 2009). Transported from nucleus to cytoplasm with the help of Exportin-5 and RAN-GTP, pre-miRNA is further processed by Dicer and Loquacious to produce miRNA/miRNA^∗^ duplex, which will be loaded into the Argonaute (Ago) by HSP70/HSP90 chaperone machinery to form mature RNA-induced silencing complex (RISC) after strand selection (Bartel, 2004; Kim et al., 2009; Iwasaki et al., 2010). Recently, miRNAs have been explored in the regulation of sesquiterpenoids. In *Blattella germanica*, silencing the expression of *Dicer-1* shows that miRNAs regulation is related to metamorphosis (Gomez-Orte and Belles, 2009), and treatment of methoprene on *Drosophila* S2 cells also reveal the differential expression of miR-34, miR-100, miR-125, and let-7 (Sempere et al., 2003).

In many insects, miRNAs have also been found to potentially regulate different sesquiterpenoid pathway genes (Table 2). For instances, in mosquito *A. aegypti*, four JH biosynthetic enzyme genes including *3-hydroxy-3-methylglutaryl-coenzyme A reductase* (*HMGR*), *diphosphomevalonate decarboxylase* (*PP-MevD*), *aldehyde dehydrogenase* (*ALDH*), and *farnesyl-pyrophosphate synthase* (*FPPS*) were *in silico* predicted to be potentially regulated by miRNAs (Nouzova et al., 2018). In addition, in the adult female mosquito, mosquito specific miR-1890 targets JH-controlled chymotrypsin-like SP, *JHA15* that involve in the regulation of blood digestion, ovary development and egg deposition (Lucas et al., 2015).

**TABLE 2:** Published studies of potential microRNA regulators on insect sesquiterpenoid pathway genes.

Species	Target	miRNA	Validation methods	References
*Ae. aegypti*	*HMGR*	miR-31–5p	*In silico* prediction	Nouzova et al., 2018
	*PP-MevD*	Bantam-3p, miR-34-5p	*In silico* prediction	
	*ALDH*	miR-34–5p	*In silico* prediction	
	*FPPS*	miR-9a-5p, miR-317-3p	*In silico* prediction	
*An. gambiae*	*JHAMT*	miR-278	*In vitro*	Qu et al., 2017
	*Met*	miR-8, miR-14, miR-34, miR-278	*In vitro*	
*Dr. melanogaster*	*JHAMT*	Bantam	*In vivo*	
	*JHAMT*	miR-252, miR-304	*In vitro*	
	*Gce*	Let-7, miR-8, miR-14, miR-34, miR-278, miR-304	*In vitro*	
*Tr. castaneum*	*JHAMT*	bantam, miR-252a, miR-304, let-7, miR-92b	*In vitro*	
	*Met*	miR-92b	*In vitro*	
	*Met*	miR-6-3p, miR-9a-3p, miR-9d-3p, miR-11-3p, miR-13-3p, miR-13a-3p, miR-2944a-3p, miR-2944b-3p, miR-2944c-3p, miR-3804a-5p, miR-3893-3p	*In silico* prediction	Wu et al., 2017
	*Kr-h1*	miR-6-3p, miR-9a-3p, miR-11-3p, miR-13-3p, miR-13a-3p, miR-2548-3p, miR-2944a-3p, miR-2944b-3p, miR-2944c-3p, miR-31a, miR-31b-5p, miR-31c-5p, miR-3893-3p, miR-6531-5p	*In silico* prediction	
*Lo. migratoria*	*Kr-h1*	Let-7, miR-278	*In vivo*	Song et al., 2018
*Bl. germanica*	*Kr-h1*	miR-2 family (miR-2, miR-13a, and miR-13b)	*In vivo*	Lozano et al., 2015
*Da. pulex*	*JHAMT*	Bantam, miR-92, miR-252b	*In vitro*	Qu et al., 2017
	*Met*	Bantam, miR-278	*In vitro*	
*N. denticulata*	*JHAMT*	Bantam, miR-92, miR-252	*In vitro*	
	*Met*	miR-8, miR-34, miR-278	*In vitro*	
*S. maritima*	*JHAMT*	Let-7, miR-34, miR-252, miR-278	*In vitro*	
*Ta. tridentatus*	*JHAMT*	Bantam, let-7, miR-34, miR-92, miR-278	*In vitro*	
	*Met*	Bantam, let-7, miR-8, miR-34, miR-252	*In vitro*	

*For details, please refer to the text.*

In *T. castaneum*, developmental defects and lethality are observed after knocking down *Dcr-1* and *Ago-1*, and *in silico* prediction showed that putative JH receptor *Met* and JH-inducible transcription factor *Kr-h1* were targeted by 11 miRNAs and 14 miRNAs respectively (Wu et al., 2017).

In *L. migratoria*, Ago-1-dependent miRNAs are involved in oogenesis (Song et al., 2013), with let-7 and miR-278 caused decrease of yolk protein precursors results in defects of ovarian development and oocyte maturation through *Kr-h1* (Song et al., 2018), and application of miR-2/13/71 agomiR leads to inhibition of oocyte maturation and ovarian growth whilst the expression level of this miRNA cluster could be decreased to achieve vitellogenesis and oogenesis (Song et al., 2019).

In *B. germanica*, expression of Dicer-1 whose depletion causes sterile females, is negatively related to JH levels, indicating the important roles of miRNAs and interaction between miRNAs and JH in oogenesis (Tanaka and Piulachs, 2012). Specifically, treatment with miR-2-inhibitor on last instar resulted metamorphic defects, and treatment with miR-2 mimic on the Dicer-1-depleted juvenile can complete metamorphosis from nymph to adults (Lozano et al., 2015).

In order to strengthen ability of adaptation, brown planthoppers, *Nilaparvata lugens*, shows polyphenism with two phenotypes, long-winged and short-winged morphs. miR-34, whose expression level can be upregulated or downregulated by JH and 20E, respectively, can target insulin receptor-1 to be involved in the modulation of wing polyphenism (Ye et al., 2019).

In *H. armigera*, 20E and JH are involved in the control of climbing behaviors of single nucleopolyhedrovirus (*HaSNPV*) infected larvae. Methoprene treatment decreases expression of *Br-C Z2* and increases expression of these miRNAs miR-8 and miR-429 which could target *Br-C Z2* (Zhang S. et al., 2018), implying the miRNA-mediated crosstalk between 20E and JH.

**FIGURE 9:**
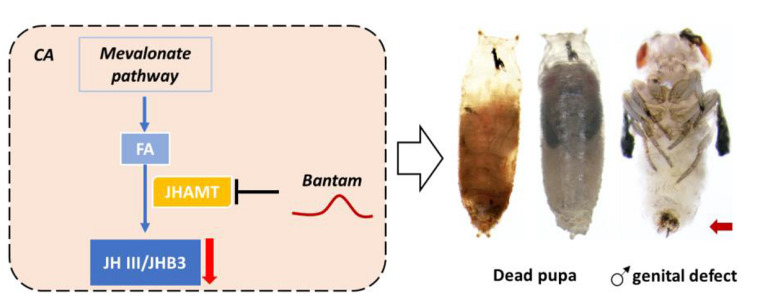
MicroRNA bantam regulates JH titer via targeting *JHAMT* in *D. melanogaster*. Up-regulation of bantam repressed expression of *JHAMT* and reduced the titer of JH III and JHB3, which resulted in dead pupa and male genital defects.

In *Drosophila*, miRNA *bantam* has been found to interact with *JHAMT* both *in silico*, *in vitro*, and *in vivo* (Qu et al., 2017). The overexpression of microRNA *bantam* in the brain decreases expression levels of JHAMT.; The knockdown of *bantam* increases the expression level of *JHAMT* (Qu et al., 2017; Figure 9). Hormonal measurement in *bantam* mutants demonstrates decreased sesquiterpenoid levels and male genital defects. *bantam* mutant phenotypes can be rescued by exogenous sesquiterpenoid application (Qu et al., 2017). In other arthropods including other insects, crustaceans, myriapod and chelicerate, the roles of *bantam* and other miRNAs on *JHAMT* and *Met* have also been tested both *in silico* and *in vitro*, revealing a conserved system of miRNAs in regulation of sesquiterpenoids established in the arthropod ancestor (Qu et al., 2017; Table 2). A list summarizing the latest knowledge on miRNA regulation of sesquiterpenoid pathway genes are shown in Table 2.

## Author Contributions

SSKT, SL, CL, and JH wrote the first draft of the manuscript. All authors proofread the final version of the manuscript.

## Conflict of Interest

The authors declare that the research was conducted in the absence of any commercial or financial relationships that could be construed as a potential conflict of interest.
